# Child-Murder in Its Sanitary and Social Bearings

**Published:** 1858-07

**Authors:** William Burke Ryan

**Affiliations:** Fothergillian Gold Medallist for an Essay on "Infanticide in its Medico-Legal Relations."


### CHILD-MURDER IN ITS SANITARY AND SOCIAL BEARINGS.

By WILLIAM BURKE RYAN, M.D.Lond.;
Fothergillian Gold Medallist for an Essay on "Infanticide in its Medico-Legal Relations."

In order that the sanitary and social inquirer, whose business it is to prevent untimely death in any shape, and to whom the question of infanticide peculiarly belongs, may properly appreciate this grave subject, it will be necessary to recall to his mind the extent to which it prevailed in different ages of the world's history, and to glance at causes which have influenced deeds that must appear, at least to Christians, as a cruel war with Nature and a sin of the deepest die. I consider it utterly impossible properly to treat of infanticide without viewing it in all its moral bearings; for assuredly it is of the most vital importance to a Christian nation to eradicate, if possible, the smallest tendency to such a crime from the minds of its people. I say, to a Christian nation, because all, or almost all, Pagan nations and peoples, who were uninfluenced by feelings of accountability on the matter, either observed the practice as part of their codes of laws or followed it from feelings of superstition, as an affair of convenience, from the pressure of want, or even from fashion. And facts and figures, in which the sanitarian delights, prove incontestably that Christianity has done more than any other institution—nay, than all institutions put together—to stem the torrent of infanticide.

There is a growing feeling of uneasiness abroad upon the subject of infanticide; the popular mind is disquieted by the conviction that our land is far from blameless, nay, is most culpable, in respect to this crime. There are few sections of the community, if they rightly direct their minds towards it, that may not lend a hand to put it down; but the medical profession, from the habits, the influence, and the opportunities of its members, is particularly powerful. Let them never forget their mission!

Infanticide,* from whatever motives, presents murder in one of its most revolting forms, whether perpetrated wholesale, as in ancient and modern Pagan nations, or in sometimes more than isolated cases in civilized communities.

Before and after the deluge, the heathen gods, or demons, had human sacrifices offered to them, especially of the children of those sacrificing; and we learn from Sanchoniatho, Pau-

* Lycophron is the authority for the Grecian deity being called *Infanticida*, which was thought to have been applied to Hecate or Diana in one of her other forms.

sanias, and other ancient authors, that such sacrifices were common in Phœnicia and in Egypt. The ancient kings of Tyre offered their sons in sacrifice, and the ancient Syrians sacrificed to Jupiter and Juno, destroying their children without mercy.

We do not find any specific punishment in the laws of Moses for infanticide, and reasons exist to make us infer that the crime was unknown amongst the Jews. The expectation of the Messiah must have acted as one great cause, and besides, as remarked by Dr. Priestly, the purest morality was the principal object of the system of Moses. Tacitus, with all his enmity to the Jews, acknowledges that no man was allowed to put his children to death.* I am informed, by a Jewish friend, that the detestable crime is almost unknown amongst the Jews of the present day, This, equally with its prevalence in Pagan countries, is a great fact for the sanitary observer.

When the Jews, however, freed themselves from the law, and held intercourse with the surrounding idolatrous nations, amongst whom immolation of children at the shrines of their deities was most extensive, they erred grievously in this respect and sacrificed their sons and daughters.

The practice of burying children alive in ancient Persia was, according to Herodotus, common. The same author says, it did not exist in Egypt in his time, but there is little doubt that the practice at one time existed. The proofs afforded by Sir John Marsham and Bishop Cumberland induce Whiston to believe that human sacrifices were common. It is said that the practice of abortion and infanticide, particularly the former, prevails very much at the present day, and that there are professors for that purpose among the women. At Cairo Arabian physicians follow the horrid practice as a profession.

The Greeks, with the exception of the Thebans, quickly disposed of their children, either by exposure or death. The Thebans held the crime in detestation, and made its committal a capital offence—offering, in this respect, a marked contrast to the Athenians.† State reasons chiefly influenced the practice, weak and sickly children being considered as likely to be of no use. Some, as the Pelasgians, offered every tenth child as a sacrifice of propitiation in times of scarcity.

The Carthagenians, like their ancestors of Tyre, immolated children, and Gelon, king of Syracuse, as well as the Roman people, endeavoured to stop these sacrifices. On being besieged

* Nam et necare quemquam ex agnatis nefas.

† "Contra morem legesque reliquorum Græcorum et imprimis Atheniensium." (ÆLIAN.)

by Agathocles, they sacrificed two hundred of the children of their most illustrious families.

The nations of the North, the people of the Baltic, Servians and Scandinavians especially, sacrificed to Thor and Woden.

In early Rome, paternal authority was sanctioned in almost its highest degree, the father having all but absolute authority over his children. Exposure and slaughter of children were rife; these being abandoned and destroyed in various ways,—sometimes given up to wild beasts, sometimes thrown into rivers. The absolute "patria potestas" was restricted under the emperors.

That licentiousness, in progress of time, was extreme, we learn from Juvenal and the other satirists and authors of the period, as well as that the practice of abortion was very frequent. In the time of Constantine the practice of infanticide was very prevalent and on the increase, owing apparently to distress; and on this emperor becoming Christian, he caused laws to be enacted, whereby funds were allotted out of the public treasury to parents overburthened with children, in order to take away the temptation of suffocating, or exposing them to be sold; and also according the rights of property in the exposed children to those who had the charity of taking pity on them.

During the fearful persecutions to which the early Christians were exposed, calumnies of all sorts were unsparingly heaped upon them, and amongst the rest it was asserted that they devoured children at their meetings. This groundless calumny was triumphantly refuted by the Christian fathers of the day, as well as by Minucius Felix, a lawyer (cap. xxx, Oxon. 1627, p. 98), who boldly hurled back the calumny on the heads of their accusers.

Although Tiberius, in the third century, tried to put a stop to the practice, and caused the pagan priests who presided at the sacrifices to be hanged upon crosses made of the trees that shaded their temples, it approached the fifth century before it was put down.

Thus, then, we see that the murder of children was very general in ancient times; that it was palliated and excused on grounds of necessity, and allowed by lawgivers and philosophers of different countries. We see, as is acknowledged by Gibbon, that the destructive flood was first stemmed by the gentle influence of the Christian religion, which even in its infancy tended to arrest horrors so rooted and so general; as well as that this influence was insufficient until "fortified by the terrors of capital punishment."

It is stated by writers that the Mahommedan invaders of many countries did, at all events, one service, that of prohibiting child-murder. This one good, remarks Colonel Walker, resulted from the imposture of Mahomet; and certainly the Koran expressly forbids the crime:—"Kill not your children for fear of coming to want; we will provide for them and for you: verily the killing of them is a great sin." Sir John Chardin, however, says that the Mahommedan Tartars, when they cannot maintain their children, think it a charity to murder them when newly born, as well as when sick and past recovery. Many other authors show that they allow the practice, but that its frequency is lessened by the universal custom of procuring abortion, a crime so strictly of a pagan character.

Our peculiar relations both to India and China make the subject of infanticide in these countries of great interest to us, particularly as regards the former, on account of the great, and in many respects successful, efforts of the English nation to stop the practice there.

Hindostan appears to have been some time since the great field of slaughter of female infants. This was particularly the case among the tribe of Raj-Kumar, which deduced its descent from the ancient princes of Delhi, and numbered about 40,000. The shame felt upon being unable properly to apportion their daughters in marriage is said to be the chief reason, and the modes of causing death are various. The custom of drowning female infants exists in Kutch, in the peninsula of Guzerat, among the Kalowries of Scinde and others. The custom does not descend to illegitimate children, as they will not have to portion such as these. So savage are some of the women, that even when married to Mahommedans they continue the practice, and this against the religion and wishes of their husbands, in order to advantage the tribe to which they belong. It was said, but with every appearance of exaggeration, that in *Kutch* and *Kuttawar* 20,000 were murdered annually, but it was believed that in *Guzerat* 5000 perish yearly. In Kutch, Hallar and Matsyn-Kantha, one account places the infanticides yearly at 30,000, another at 3100, but Colonel Walker says this number is as short of that destroyed as the other is beyond it.

With the Raj-Kumars and other tribes of Bengal the crime was prevalent, but has been abolished; also among the Rhatore Rajpoots of Jeypore and Jondpore, as well as among the Jaut and Mewat tribes of Hindostan. The children are dropped immediately after birth into holes filled with milk, or the placenta is put on the mouth till the child expires, or opium is put on the nipple, which the child sucks in with the milk and dies. The means taken by Governor Duncan and Colonel

Walker have been very successful in stopping this custom, and are highly creditable to them, as well as to the English nation, which empowered them to act, but it is to be feared that nothing short of the introduction of Christianity will ever permanently overcome the evil. Sir W. Sleeman shows that these murders are still very frequent.

The prevalence of at least female infanticide to a very great extent casts its dark shadow over the Chinese character. Authorities are at variance as to the number yearly murdered—cut in two or poisoned, perhaps in order to obtain a plentiful harvest, or destroyed for one reason or another. The practice of drowning is general, says Gutzlaff. In recent communications from Dr. Williams and Sir John Bowring, we find that in many parts of China there are towers where children are thrown into a hole made in the wall, and it is said, on the authority of Father Ripa, that of abandoned children, the Jesuits baptized in Pekin alone not less than 3000 yearly. Dr. Williams says he has seen ponds which are the habitual receptacle of female infants, whose bodies lie floating about on their surface. Meanwhile, their penal code prohibits infanticide. Sir George Staunton, as well as Du Halde, found exposure of children very frequent in Canton and Pekin; they estimate the number in Pekin alone at 2000. Facfur, a prince of the province of Mangi, saved 20,000 annually of children exposed through the poverty of their parents. Barrow states that from 10,000 to 20,000 annually are exposed, slaughtered, or interred alive throughout the kingdom; the Abbé Bergier places the number at 30,000. Exposed on the highways, they are either trampled to death by animals or devoured by vultures.

In Arabia female children are frequently murdered. The Niam-Niams, an African race, devour, it is said, the children of their enemies. In some parts of America, among the aborigines, Robertson says, when twins are born, one is destroyed, and when the mother dies while nursing, the infant is buried in the same grave. So also in New Holland. In Greenland, Labrador and California, children were destroyed during times of scarcity.

In Mexico, almost fabulous numbers are given by Torquemada, Zumurraga and others, as being sacrificed annually, 20,000 even being named. The Peruvians were said by Acosta to rival the Mexicans in the slaughter of children. In South America, in New South Wales and in New Zealand infanticide is common. The same may be said of some of the South Sea Islands,* Japan, Ceylon, Madagascar, and many other places which I must leave untouched.

* It is stated on good authority that in Otaheite two-thirds of the children

By infanticide, in medical jurisprudence, is understood the murder of a new-born child, but there is no specific time named to which the term "new-born" applies, the term not being restricted to days after birth. Trials in such cases are conducted as those of ordinary murder. Proof is required that the child was *wholly born* and had an existence independent of the mother. The laws have varied much in this country at different times regarding infanticide. The Act 21 James I, where a bastard child was born dead, required proof on the mother's part that the child *was* born dead—in default of which she was visited with punishment. This statute was so unjust and inhuman that it could not long remain law, especially as the punishment for the offence was no less than death, The laws against infanticide all over Europe, or nearly, were formerly of extreme severity, and the above act seems to have been adopted from the French criminal code. In France, in 1556, by an edict of Henry II, every unmarried woman who concealed her pregnancy and caused the death of her infant, was herself condemned to death. Immaturity of the child formed a sufficient defence. This law, like our own, has been modified. Immaturity in this country also forms an efficient defence, but the accused must give this proof. Maturity of the child is necessary in order to establish the crime of concealment of pregnancy, which takes the place of child-murder of former times, and is punished with two years imprisonment at the utmost. Concealment of pregnancy and birth is not often made the subject of prosecution, but where an indictment is laid for murder, and the proofs fail, although the case is very suspicious, then the law falls back upon concealment, for which the accused may be tried. In 1803 an act was passed, which provided that women tried for the murder of bastard children are to be tried by the same rules of evidence and presumption as by law are allowed to take place in other trials of murder, leaving power of a second trial for concealment if the first failed. This statute was modified by the Act 9 George IV, where on proof of concealment of birth, it was not necessary to show whether the child died before or after birth.

The stream of prejudice now runs on the other side, and there is no crime that meets with so much sympathy, often of the most ill-judged kind, as that of infanticide, and this sympathy seems to have seconded, if not guided, the law in this respect. An almost partisan feeling has often been evinced,

---

were destroyed before the introduction of Christianity, and that there were few women who had not embrued their hands in the blood of their children. So of Japan, etc.

not only by the legal, but even by the medical profession. To this must be added the great difficulties attendant on the medical inquiry as to the cause of death in these cases, but above all that almost insurmountable difficulty thrown on the medical witness, which requires him to prove whether, in case of death, the child has been *wholly born* when the violence had been committed. All the other obstacles in the way of proving that a child lived are bad enough and difficult enough, but yet legitimate subjects of medical inquiry; but this last seems purposely left open by the law, a back door for the escape of the guilty mother. It is not sufficient that the medical witness prove the child to have breathed at or about the time of birth—it is absolutely necessary, under the present state of the law, to prove that the child was alive when *wholly born,* that life existed after the body came into the world, as shown by the state of the lungs, etc.; so that in truth the killing of the child *during birth,* although it breathes and lives, is not murder *in law;* and although injuries of a deadly character may appear, yet unless a medical man can prove that the child was *wholly born* when it was killed, the accused must be acquitted. That this is the state of the law has been frequently ruled. Baron Gurney stopped a case when he elicited from the medical witness that a child might breathe during birth; and it was ruled by Mr. Justice Coleridge, at the Norfolk spring assizes, 1837, that the whole body of the child must be born in order to have an indictment for child-murder stand. Mr. Baron Parke has gone even further. He said, at the Herts Lent assizes, 1841:—"With respect to all these cases, there is a degree of doubt whether the infant has been *born alive.* The law requires that this should be clearly proved, and that the whole body of the child should have come from the body of the parent. If it should appear that death was caused *during delivery,* then you will not find a true bill."

In the *Provincial Medical and Surgical Journal,* April 23d, 1845, is reported an extraordinary case where Mr. Justice Erle laid down the law and distinguished between medical and legal life. In this case it was shown that the child had breathed, and its head, when found, was nearly cut off. The jury were told that before they returned a verdict of "Guilty," they must be satisfied that the child had an existence distinct from, and independent of, the mother; that it was, in fact, *wholly born* when murdered by her. That the child might have respired before it fully came into the world, and that although such might be, *medically,* a live child, yet that it was not so *legally.* Of course the jury acquitted the prisoner.

Hear the following case. At an assizes, 1855, Jane Perry was charged at Gloucester with the wilful murder of her male infant. On the surgeon entering the room, she was in bed with her head on the bolster, underneath which was a dead child with its throat "divided by some instrument," but the surgeon could not say positively whether the child had been *born alive.* In consequence, Mr. Baron Martin said the prisoner could not be convicted of murder, and the jury in consequence found her guilty of endeavouring to conceal the birth of the child.

I need scarcely say that this state of the law throws a great responsibility on the medical witness, I may add a responsibility which should not be thrown upon him ; for it is generally utterly impossible for him to give the proof required ; and in fact, once that he has given proof that the child was alive at the time that violence was offered to it, *there his duty, as well as his power*, seems to cease, as it is almost as much in the power of non-medical as of medical witnesses to say whether the child was or was not *wholly born* when violence was offered. Some additional circumstances, some corroborating witness must be looked for in order to the conviction of the accused. The medical witness has to depose to certain facts and to answer certain questions—beyond that his jurisdiction ceases, and he can show no leaning one way or other ; the jury hold the scales, and they must determine.

The present state of the law seems equally unfair towards the jury. They have no alternative but to convict the accused of murder or to acquit her, and there are but too often, unfortunately, in the circumstances of the unhappy woman tried for her life facts that appeal to their commiseration. Seduction, perhaps with desertion and misfortune of one kind or other, plead, and properly plead, on her behalf, and a verdict of "Wilful Murder" is scarcely ever returned, but the jury fall back upon the power left them and return a verdict of concealment of birth. But often this verdict does not appear commensurate to the amount of offence proved, and it appears desirable that a modification of the law should take place as regards the crime. The crime of infanticide is but too frequently accompanied by the most determined and cold blooded calculation, and where this great repugnance to capital punishment is felt, juries and judges should have much stronger powers than they have at present. The law in France would seem to meet this point by finding for the capital offence "with extenuating circumstances." This would enable the judge to measure out the punishment according to the nature of the conduct that accompanied the crime, and might generally meet all the requirements of justice. "Two years imprisonment," says Dr. Taylor, "is substantially the punishment at present inflicted for the crime of infanticide in this country; for it is not to be concealed that, *medically* speaking, these technical points relative to 'live birth,' to 'entire birth,' or 'to an independent circulation in the child,' or lastly, 'to concealment of birth,' are only so many ingenious means for evading convictions on the capital charge." A modification of the law like that alluded to, Dr. Taylor adds, "appears to be necessary, unless we are prepared to admit that the destruction of a living and breathing child *during* the act of birth is not a crime." May it not be asked what a mockery of justice is this state of the law, what a tampering with life, and what an inducement to murder? Every man, perhaps, of a jury believes a prisoner guilty of murder, but because they have not proof that the child was *wholly born*, that is, entirely separated from the body of the mother *when it was killed*, they acquit her. This state of the law is no secret to some of those who intend to benefit by it, and perhaps no more important subject of legal medicine can be brought before the profession and the public for calm and impartial reconsideration. The laws of Prussia are pretty much on a par with our own as regards infanticide, and the difficulties in the way of conviction, as well as the unwillingness of juries to convict, are very similar.

In March 1857, Lord Raynham moved in the House of Commons for a return of the number of persons convicted of infanticide from 1852 to 1856 inclusive, together with the sentence passed upon each; if modified or reversed; and specifying also whether the verdict had been accompanied by a recommendation to mercy, and if so, the reason or reasons, if any, alleged, etc. Returns not yet printed have been made, but they give very little information. The strange fact, however, appears in some of them that the clerks of assize had no means of answering whether the sentences had been modified or reversed; as well as that it was not customary to minute the jury's recommendation to mercy. One clerk says, "I hardly know what is intended by the word *Infanticide*."*

The return made by the clerk of assize of the Home circuit in these years is marked "*none*."

---

* It will be here also remarked, that the returns give only the *convictions* for infanticide—not the absolute trials, much less the trials for concealment of birth. Proper returns from Coroners' Courts are what we are most in need of; these courts are the true sources whence to build up the statistics of infanticide. Such returns were moved for in the House of Commons in 1852, but never made.

The *only* case of a conviction for the murder of a child was at the Surrey Lent assizes, 1852. The punishment is not specified; nor is it in another case at the same place, where a woman was convicted of manslaughter on an indictment charging her with infanticide.

In the Midland circuit, 1853, Mary Ann Parr had sentence of death passed upon her at Nottingham, but it was commuted to transportation for life. Ann Tommey was indicted for wilful murder at Warwick, 1853; found guilty of manslaughter. The sentence twelve months imprisonment with hard labour. At Lincoln, 1855, Elizabeth Lownd was indicted for wilful murder. She was transported for fifteen years. At Derby, Eliza Beastall was indicted for a similar offence, and was imprisoned with hard labour for two years.

*Northern Circuit.* Jane Gillie: sentence of death recorded;—punishment, transportation for life.

*Norfolk Circuit.* One woman sentenced to death;—punishment, transportation for life.

In Lancaster, one indictment; result not stated.

*Oxford Circuit.* 1. Abel Ovens: child six weeks; sentence, to be hanged;—punishment, transportation for life. 2. Eliza Dore: similar sentence, similar commutation. 3. Mary Robins: ditto, ditto. 4. Sarah Baker: child two years; ditto, ditto. 5. Maria Tarrant: child three months; ditto, ditto.

*Western Circuit—Cornwall.* Richard Jose, jointly with the mother and another person: verdict, manslaughter;—punishment, transportation for life.

*South Wales.* None.

*Chester and North Wales.* 1852. Margaret Davies, county Denbigh: convicted of murder; sentence, death;—result not stated.

*County of Chester.* 1. Indicted for murder; conviction, manslaughter; sentence, seven years transportation. 2. Indicted for murder; convicted of murder; sentence, death;—commuted to transportation for life. 3. Indictment for murder; convicted of murder; sentence, death;—punishment, transportation for life. 4. Same indictment; same conviction; same sentence;—result, transportation. 5. William Jackson: murder of his two children, five and seven years of age; same indictment; same conviction; same sentence;—result, hanged.

That the crime of infanticide, as well as that of criminal abortion, is wide spread and on the increase, is, I fear, but too certain. We have only to look to the daily or weekly papers to be painfully convinced of this fact, as well as that it will require a strong hand to put it down.

"The 'Circuit Calendars'", says the *Legal Examiner* 1853, "exhibit, as usual, a number of cases in which infants have met their deaths at the hands of their mothers." It adds that "there is a horrible resemblance between the nature of the circumstances attending these deaths; but that there is one difference—that they are more numerous than they used to be. The public, as well as the judge and the bar, notice it; and Mr. Justice Coleridge pointed it out to the grand jury at Worcester." "We shall soon", continues the *Examiner*, "rival the Chinese people in callousness to infant life."

See what inquiry brings forth amongst a Christian people. Look at the *Sanitary Inquiry Report*, Supplement, "Interment in Towns, 1843", by Edwin Chadwick, Esq. In the Manchester and Salford district, the minister was often shocked by a common phrase amongst the women of the lowest class, in alluding to children—"Aye, aye, that child will not live; *it is in the burial club.*" The actual cost of a funeral being from £1 to 30*s.*, and the allowance from clubs in the above place being from £3 to £4, and even £5, a balance is left to tempt the demon parents. Moreover, it was customary to insure a child in four or five of these burial societies; and it was stated that one man had actually insured such payments in nineteen different burial clubs in Manchester.

Mr. Gardiner, clerk to the Manchester union, deemed the cause assigned by a labouring man unsatisfactory, and refused to register a death. On inquiry, he found rumour had attributed the death to wilful starvation. The child had been entered in *at least ten burial clubs;* and the parents had six other children, who only lived from nine to eighteen months respectively. They had received £20, from several burial clubs, for one of these children; and they expected to receive as much for this child. The child had no medical care. The jury thought the evidence of the parents made up for the occasion, and not entitled to credit. The verdict was, "Died through want of nourishment; but whether occasioned by a deficiency of food, or by disease of the liver and spine, brought on by improper food and drink, or otherwise, does not appear." After this verdict, the man enforced payment from ten burial clubs, obtaining £34 : 3 : 0.

Two similar cases came under the notice of Mr. Coppock, of the Stockport union, in both of which he prosecuted the parties for murder. In one case, where three children had been poisoned with arsenic, the father was tried with the mother, and convicted at Chester. He was transported for life: the mother was acquitted. In the other, although the judge summed up for a conviction, the accused, the father, was to the astonishment of every one acquitted. The body of the child was afterwards exhumed, and arsenic found; as was also in the bodies of other two children of the same man. In all these cases, payments were enforced from the burial clubs. It was remarked of these dreadful cases by the superintendent-registrar, that the boys, as being likely to be of use to the parents, were spared; while the female children were the victims. It was the clear opinion of the medical officers that infanticides were committed in Stockport in order to obtain money. A Liverpool woman named Eccles was convicted of the murder of one child, and was under the charge of poisoning two others with arsenic. Immediately after the murders, she went to demand of the burial societies a stated allowance. Cases of culpable neglect of children who were thus insured had been observed at Preston. The collector of one of the most respectable burial societies in Manchester had strong grounds for believing that it had become a practice to neglect children for the money allowed. A vast number of frauds have been perpetrated on the different societies in this manner.

"I have no doubt", says the town-clerk of Stockport, writing in 1843, "that infanticide to a considerable extent has been committed in the borough of Stockport." He prosecuted in two distinct charges; one victim being sixteen years of age, and unlikely from weakness to be serviceable to her father. An inquest was held, and a verdict of natural death returned. In three months afterwards, the body was exhumed and a very large quantity of arsenic detected. Mr. Justice Coleridge summed up for a conviction; but the jury acquitted the prisoner. A verdict so extraordinary, it was remarked, could only be accounted for by the general feeling against capital punishments, which enables so many criminals, capitally indicted, to escape punishment. This man received £8 from burial societies, and freed himself from the burthen of the poor girl's support. The other case involved no less than three distinct cases of murder. Robert Sandys and George Sandys, with their wives, were the accused, their own children being the victims. They were all in burial societies.

Dr. Granville, in his work *Sudden Death*, compiled from the Annual Reports of the Registrar-General, thinks the vast mortality amongst children must be looked upon with great suspicion. He says: "Frightful as this early destruction of human life must seem in the abstract, I grieve to add that, as we come nearer to the present times, not only does the general amount of life thus extinguished, as it were, on its threshold, increase, but the increase appears under circumstances capable of inspiring grave suspicions of its not being altogether natural. Thus I find that the early destruction of life is greater in certain manufacturing districts than in purely agricultural localities." In alluding to verdicts of "found dead", "accidentally overlaid", "suffocation from having taken an over quantity of mother's milk, she having fallen asleep while the child was sucking", and such like, he says: "Now, in all cases where the preceding verdicts were delivered, either the children were illegitimate, or the parents steeped in poverty," etc. He thinks that the large mortality of children under one year throughout England and Wales during the years 1847-8-9 (267,086), calls for some serious investigation into its origin and causes. He adds: "And let the legislator and moralist look to it; for, as sure as there is in any nation a hidden tampering with infant life, whether frequent or occasional, systematic or accidental, as in the cases of the poisonings in Essex, or the burial-club iniquities, so sure will the chastisement of the Almighty fall on such a nation. . . . In some parts of England", he adds, "the manufacturing towns, to wit, such as Manchester, Ashton, Preston, Leeds, etc., this early mortality may be rightly called frightful."

"The natural instinctive horror of blood", says the *Morning Chronicle*, "the reverential sense of the sacredness of human life, seems to be becoming extinct among the humbler classes."

Mr. Hilles, in the *Legal Examiner*, considers that the crime of infanticide has spread to a fearful extent, and seems to be extending rapidly amongst the humbler classes of society. He doubts whether the closing of Foundling Hospitals in this country has not been more injurious than otherwise to public morals.

The most exaggerated statements take place as to the number of exposures and infanticides in London alone; and I saw some time since, in a medical publication, the number placed at eleven hundred: but, from other and surer sources, we can learn that the amount of murders is very great. Thus, in the papers of about three months since, Mr. Coroner Wakley is reported to have said that three hundred deaths by infanticide take place annually in London, although many are returned as dying from other causes, "still-born," etc.; adding, that with ten thousand pounds at his command, he could put a stop to the iniquity. Surely would freedom from such a system be cheaply purchased at a much larger sum. As a commentary on this, in the Registrar General's Weekly Return for about the third week of April last past, six deaths are given as by "suffocation"—*five of these being infants.* The death of none of these may have been caused by foul means, but when such numbers often meet the eye, they must give rise to suspicion.

With regard to the crime as it was said to exist under the burial-club system, a person should think that no commiseration could be entertained towards parents who could coolly plan and plot, day after day, the murder of their offspring. Sympathy for that class would be false indeed, and only tend to encourage them in their bad tendencies.

The case is different with an unfortunate girl, maddened perhaps by overwhelming shame and a sense of wrong, and all excuse that can be made for the deed of a woman in such a position, short of excusing actual murder, she should have the benefit of; but as long as the laws of the country treat the crime of infanticide as they do at present, the medical witness will often have a difficult task to perform. He cannot become the partisan of any criminal; but having a sacred duty to discharge, he will have to sift the circumstances, extract the truth and depose to it, and then leave to the juries of the country the task of pronouncing as to guilt or innocence. He will not fail in these, as in all other cases where there is any doubt, to do all he can to have the accused get the benefit of it, but he will not strain truth for this purpose. He will recollect that there can be no crime of a more dreadful nature than murder, and that no excuses beyond those that can be truthfully made should be made on behalf of any one guilty of the crime. The great obstacle thrown in the way of justice in these cases consists in the fact that it is not sufficient for the medical witness to prove that the child breathed at or about the time of birth. The present state of the law renders it absolutely necessary to prove that the child was alive when *wholly born*—that life existed after the body came into the world. So that in truth the killing of the child *during birth* is not considered murder in law. A judge has told a jury that if they were of opinion that the prisoner had *strangled her child* before it was *wholly born*, they were bound to acquit her! O, wise laws! This is a strange and anomalous state of things, but the medical witness must take the law as he finds it; he must not go beyond it. There seems too much of fashion in this unwillingness on the part of juries to convict, and it would be well if it were done away with. Look at the inconsistency of juries, as shown by the following case from the papers, where a poor woman, *already dead* from "epilepsy produced by exhaustion," is brought in guilty of wilful murder of her infant. Had the woman lived, I presume she would have been acquitted!

CONCEALMENT OF BIRTH, AND VERDICT OF WILFUL MURDER.

—On Tuesday, an inquest was held at the Calcutta Inn, Gloucester-road, Cheltenham, by J. Lovegrove, Esq., on view of the body of a young woman named Mary Anne Brunsley Gilkes, and on the body of a male child, of which she had been delivered shortly before her death. The case is peculiar, from the fact that the birth of the child was not discovered until after the interment of the mother; and from the medical gentlemen who saw the young woman shortly before her death having given a certificate that she died from epilepsy, produced by exhaustion, no suspicion having been entertained by any one that she had been delivered of a child. Mr. Lovegrove, after the discovery of the infant, issued an order for the exhumation of the body of the mother, and for a *post mortem* examination of both bodies. After the jury had been to the Cemetery dead-house to view the body of the child, the evidence was taken with respect to both cases, and at its conclusion the jury returned a verdict that the deceased Mary Anne Brunsley Gilkes had died from epilepsy, arising from exhaustion, through loss of blood and neglect in her confinement; and that the child had died through the wilful design or neglect of the mother, who was therefore "guilty of wilful murder."

Let the innocent by all means be protected, and let none be visited with, at all events, the severest form of punishment short of death, unless where murder is most clearly proved. Better a thousand guilty should escape than that one innocent should suffer. But when things are fully brought home—when a cool and premeditated murder is proven, then severe punishment should follow; no false delicacy should tempt the public to connive at infant murder. Let no murderess be made a heroine of, and the practice may be lessened. Above all, let women feel that they should not be visited with so much indignation for simple pregnancy as for murder: the one is a sin of the passions, and may be momentary; the other is but too often coolly premeditated;—and if the state of morals cannot be brought to that degree of purity as to prevent illicit intercourse, they can at all events be brought to a state when a woman will long hesitate before she imbrues her hands in innocent blood. Let society, if possible, look on the fact of illegitimate pregnancy with a more forgiving eye, and pity, at all events, the unhappy victims of seduction, or the otherwise innocent who may have fallen. Let such victims have their future course through life of a less hopeless character; let them feel that an occasional flower may be scattered on the thorny path that lies before them, and that a green spot may now and then glad their eyes and give rest to their limbs; that life is

N 2

not to be of so wholly unendurable a character as they may suppose; that it may no longer be from their own sex, that—

"Every woe a tear may claim,
Except an erring sister's shame,"

and much may be done to lessen the evil. The moral criminality of such deeds should by every possible means be kept steadily before the people. This would, indeed, be a meritorious work to undertake.

I incline to the belief that the existence of Foundling Hospitals would conduce much to the prevention of the crime, but there is neither space nor time to enter at length upon the merits or demerits of these institutions. The great points that they may be abused, as when married parents, too lazy to support, wish to desert their children; or that they lead to greater immorality as to sexual intercourse, I consider light in the scale, if they only put a stop to the more monstrous vice—the more crying evil. I can well imagine that greater laxity of morals as to this intercourse may lead to more illegitimate births in countries where they exist, but I cannot believe that infanticides are likely to occur in equal numbers where hospitals are supported, as in those countries where they do not form part of the institutions. Such an argument for the depravity of human nature I may well hope cannot be sustained; indeed it would be against all past experience to come to such a conclusion, for it was in order chiefly to prevent this crime that Foundling Hospitals were instituted and kept up by some of the greatest philanthropists of former times.

It is melancholy to reflect that in the ancient days of Rome, many who took to their homes the poor children that their parents made outcasts, did so from a most sordid self-interest, as they often put out their eyes or maimed them in different ways, in order that by exhibiting them in this condition they might induce the charitable to give them money.*

With very different feelings did those who instituted Foundling Hospitals go to work. Their sole object—men and women—seemed to be to put a stop to infanticide, and by throwing the mantle of charity and secrecy over the fallen woman, and showing, by pointing out a mode of support for her child, that her whole prospects in life were not to be ruined by having a dead weight upon her exertions, they took away, once and for ever, the greatest incentives to infanticide. As in the conducting of every other institution for the amelioration of human

---

* Even Seneca vindicated the maiming of children, on the ground of their being slaves. Justinian, A.D. 530, prohibited the slavery of these unhappy ones.

suffering, it was impossible that such institutions should not be abused. They were abused, and in consequence reformations of various kinds and at different times were had recourse to, and even suppressions were tried, until it was found that infanticides increased in consequence.

A Foundling Hospital was established at Milan as early as the year 787, and one was founded, at least for orphans, in Constantinople, in 1096, by Alexius I. In 1198, Innocent III allotted part of the hospital of Spirito Santo at Rome for the reception of foundlings, in order to prevent infanticide. Austria, Spain, Russia, Belgium, etc. have many such institutions. In France they exist in greater numbers than in any other country, and great care has been bestowed upon them, as well by those who founded them and cultivated them from feelings of pure and absolute charity from the period of 1204, when Guy of Montpellier instituted one, to the time when Napoleon looked to them as a training ground for soldiers to replenish his army, and placed them under the protection of the state in 1789. At one period, as mentioned, they were suppressed in consequence of abuses; and in the reign of Louis XIII, the prevalence of infanticide caused a pious woman, Madame Legras, to institute an asylum for outcasts. She was followed by St. Vincent de Paul, whose zeal and example were all powerful in placing establishments of the kind on a firm basis.

Much has been said for and against such institutions; but the weight of evidence on the entire goes to show that they have done good and have certainly prevented infanticide. In no country where they exist as an institution can it be said, as is said of Sweden by Dr. Webster, viz., that when he visited that country recently, he found 1183 persons in the prisons, and of these not less than 106 were committed for infanticide and 26 as accessory to it, being one ninth of the inmates of these establishments! They are fostered in Catholic, while they are discountenanced more or less in Protestant countries, and it is curious to observe how the arguments for or against are influenced as they proceed from one or other of these sides.

There can be no doubt that when too great facility of depositing the children with absolute secrecy is allowed, as where the *tours* or turning boxes were established, great temptations are thrown in the way of idle, dissolute or unnatural parents to get rid of their superfluous children and throw them on the nation for support; but this as an objection could be in a great degree got rid of, and is indeed by the "*bureau ouvert*," where registration of the party applying to leave a child is made, at the same time that secrecy is observed.

The registration of illegitimate births cannot in all cases be taken as a gauge of morality, for Sir B. Brodie gives, from the Registrar General's reports, a ratio per cent. of 3·2 for London, while in Derbyshire it is 8·3, and in the North Riding of Yorkshire 9. Now, many causes may exist in London, where the standard of morality is not over high, to account for this ratio; and where, if even the compulsory "retreat of the ten thousand" public prostitutes computed to drive their trade, could be effected, there would still remain the 100,000 non-professed ones that are said to exist, from the luxurious and fashionable Calypso of the elegant suburban villa, who lures "my lord" from "my lady's" more sobered graces, to the sad and lonely child of labour whom gaunt poverty has "worn to the bone", whose fingers are benumbed by plying the profitless needle on mantles remunerating by $1\frac{1}{2}d.$ each, or on shirts or vests at a similar rate, that those may live in idleness, or worse, whose stony hearts are made stonier still by the cursed and all absorbing love of gain, and who are utterly careless how their ill got wealth increases, what wear and tear of life it causes, so that it *does* accumulate and minister to their selfishness; or for, if possible, those still more blameable, because better educated, scions of fashion who give their short orders for their gala dresses to be ready by the rapidly approaching day when they are to shine "the cynosure of neighbouring eyes", and whose robes should if it were possible be bedecked by the congealed tears—those "pearls of great price"—shed by the friendless girl just bursting into womanhood, who follows her dreary task the live-long night, till heart and soul and hope and feeling are crushed, the world appears desolate and blank, and all is chaos and bewilderment; while the tempter, it may be, steps in and secures his all but unresisting victim. The story is then soon told, and the covetous merchant and the fashionable lady have left another, albeit unacknowledged, legacy to their country.

Much, I conceive, might be effected in ameliorating the condition of women, especially of those in large towns, by laying open to them fields for industry for which they are peculiarly fitted, such as have been mooted in the watch-making movement and others of a similar nature. Women would thus be rendered more independent of general circumstances, without at the same time rendering themselves less feminine in their natures. The right woman might thus be put in the right place; and the custom would not be so general as we see it at present, as in the case of drapers' and other such establishments, of placing the wrong man in the right woman's position. This, too, would prevent the "nobler sex" being considered, as they are at present by many, less masculine in *their* natures by being put to do women's work. What valid objection can the female public entertain against employing their own sex in their own proper employments, and so adding to their independence; or what attractions have the men for them that they thus run after the man-milliners? Let an opposition-shop be tried, and the experiment may work well.

Gerando gives statistics of illegitimate births per 1,000 in countries with and in those without hospitals for foundlings.

| *States without Hospitals.* | Illegitimate Births per 1000. | *States with Hospitals.* | Illegitimate Births per 1000. |
|---|---|---|---|
| Prussia | 69 | France | 71 |
| England and Wales | 55 | Naples | 46 |
| Wales alone | 83 | Archduchy of Austria | 42 |
| Saxony | 121 | | |
| Hesse | 149 | | |

Terme and Montfalcon, Remacle, Guerry, Benoiston de Châteauneuf, etc., give many statistics regarding legitimacy in France, showing that in the beginning of the present century the illegitimates were to the legitimates as 1 in 20, at present as 1 in 14. The wars and conscriptions of the first years of the century would account for this disproportion. From 1824 to 1833, legitimate births in France were 9,031,908; illegitimate, 703,663; while the foundlings were 336,281. In 1847, the legitimate births are placed as 918,581; the illegitimate as 65,626; and the progressive increase of foundlings seems fairly attributable to increase of population.

That the mortality in foundling hospitals is great is unfortunately too true; and this has been brought forward as a strong argument against them. It is said that thus they fail in accomplishing the object in view, viz., the preservation of the lives of children. But this, I take it is not, and should not be the real object in view, which is the prevention of murder. The former object might and did influence the ancient lawgivers of Greece and Rome, with whom the benefit of the state was tantamount to all other considerations, but cannot, I presume, be acknowledged as the chief ruling motive by those who partake in the comity of present civilised nations. There is something harsh and discordant, if not horrible, in the expression of Malthus when he says, "An *occasional child-murder* from false shame, is saved at a very high price, if it can only be done by the sacrifice of some of the best and most useful feelings of the human heart in a great part of the nation." The state adopts and does all that can be done to preserve the lives of the children, yet fails in that object, and the children gradually die; *but the state does not become their executioner.* It does its best under certain unfavourable circumstances, but cannot succeed in its praiseworthy intentions. And if we take all things into consideration, the poverty of the mothers of those exposed children, the neglect to which with such mothers they should be exposed, and the thousand difficulties under the circumstances in the way of rearing them, we must leave a very large margin for mortality in such cases during the first year. Thus we find it amounts in some of the very poor quarters of Paris to 32 per cent., and in the same sort of localities in London to 33·1 and 29 per cent. if we take Lambeth (1848) and Poplar respectively. In Leeds, the mortality is 30·6; and in Preston during the strike (1853-4), the mortality was 33·3 per cent. In some parts of Russia, the mortality is 31 per cent., and of an average of 27 throughout the empire.

Tables of the mortality of foundlings in different countries at the end of the last century have been given, from which it appears that under one year the deaths were at St. Petersburgh 40 per cent.; Florence, 40; Barcelona, 60; Paris, 80; Marseilles, 90. In France, in 1824, they were 57·6 per cent.

Amongst the causes of mortality in children, many are more or less preventible. That of carrying children from a distance, especially in severe weather, ranks as one of the first; and this is aggravated in some parts of the continent, where the messengers paid for their services are not over careful of their burden. Then the various diseases incident to all hospitals; epidemics of all kinds, imperfect ventilation where atmospheric purity is so absolutely necessary, and the disease of the cellular membrane peculiar to foundling institutions, play a great part.

But the most influential of all others is undoubtedly the deprivation of mother's milk and the many imperfect substitutes for it, from the badly adapted nurse, between whom and the infant no natural tie exists, to the often equally badly adapted food. At Parthenay and Poitiers, as shown by Gaillard, where the children are suckled, the mortality is much less than at X, where they are brought up by hand; and at Lyons, where for many years all are suckled, the mortality is much less than it formerly was and than in other places less favourably circumstanced in this respect. Unnecessary detention in hospital of those intended for removal is also much to be deprecated, as greatly tending to an increased mortality.

16, Norfolk Terrace, Bayswater, June 1858.

